# Supplementary material for: Raman Spectroscopic Analysis of Highly-Concentrated Antibodies under the Acid-Treated Conditions
Source: Pharm Res. 2023 May 9;40(7):1853–64. doi: 10.1007/s11095-023-03526-9 (PMC10421790; doi:10.1007/s11095-023-03526-9)
Supplement: Supplementary file 1 — Supplementary file1 (PDF 130 KB) [file 11095_2023_3526_MOESM1_ESM.pdf]

Supporting Information

Raman spectroscopic analysis of highly-concentrated antibodies under the acid-treated conditions

Yusui Sato[1], Satoru Nagatoishi[2,3*], Shintaro Noguchi[4], Kouhei Tsumoto[2,3,5*]

[1] Analytical Instruments R&D Division, HORIBA, Ltd., Kanda Awaji-cho 2-6, Chiyoda-ku, Tokyo 101-0063, Japan

[2] The Institute of Medical Science, The University of Tokyo, 4-6-1, Shirokanedai, Minato-ku, Tokyo 108-8639, Japan

[3] Center for Drug Design Research, National Institutes of Biomedical Innovation, Health and Nutrition, 7-6-8 Saito-Asagi, Ibaraki City, Osaka 567-0085, Japan.

[4] Bio· Life Science Center, HORIBA, Ltd., 2 Miyanohigashi, Kisshoin, Minami-ku, Kyoto, 601-8510, Japan.

[5] Department of Bioengineering, School of Engineering, The University of Tokyo, 7-3-1, Hongo, Bunkyo-ku, Tokyo 113-8656, Japan.

* Correspondence and requests for materials should be addressed to N.S. (ngtoishi@ims.u-tokyo.ac.jp)

* Correspondence and requests for materials should be addressed to K.T. (tsumoto@bioeng.t.u-tokyo.ac.jp)

Figure and legend

Fig. S1

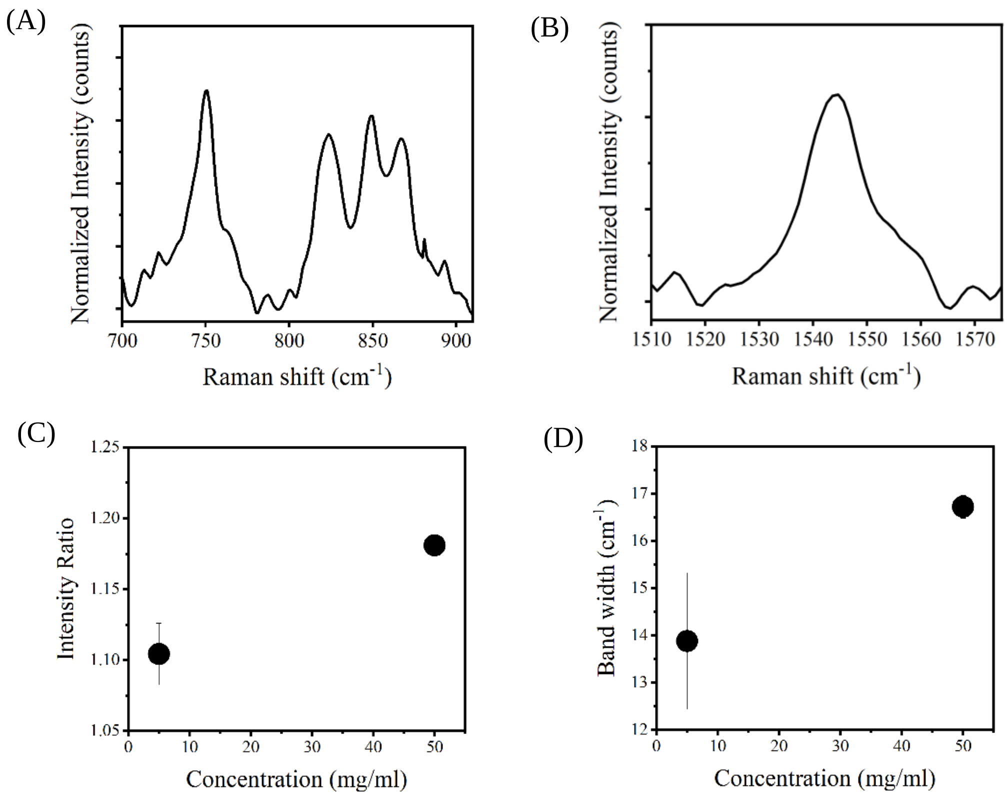

Fig. S1. (A) Tyr Raman bands at 830 and 850 cm^{-1}, and (B) Trp Raman bands at 1555 cm^{-1} of 5 mg/ml rituximab in solution of pH 7.0. (C) Plots of the band intensity ratio of I_{850}/I_{830} and (D) band width of Trp at 1555 cm^{-1} as a function of antibody concentration. The values of 50 mg/ml rituximab in solution of pH 7.0 is taken from Fig. 3. Values are means ± standard deviations derived from three independent measurements.
